# In-Hospital and Readmission Permanent Pacemaker Implantation After Transcatheter Aortic Valve Replacement

**DOI:** 10.1016/j.shj.2022.100003

**Published:** 2022-03-17

**Authors:** Michael Megaly, Ramy Sedhom, Ayman Elbadawi, Marwan Saad, João L. Cavalcante, Jay Sengupta, Santiago Garcia

**Affiliations:** a Division of Cardiology, Banner University Medical Center, UA College of Medicine, Phoenix, Arizona, USA; b Department of Medicine, Albert Einstein Medical Center, Philadelphia, Pennsylvania, USA; c Division of Cardiology, University of Texas Medical Branch, Galveston, Texas, USA; d Division of Cardiology, Brown University, Providence, Rhode Island, USA; e Department of Cardiology, Minneapolis Heart Institute at Abbott Northwestern Hospital, Minneapolis, Minnesota, USA

**Keywords:** Pacemaker, TAVI, TAVR

Transcatheter aortic valve replacement (TAVR) is an established therapy for severe symptomatic aortic stenosis and is supported by multiple clinical trials and societal guidelines. However, permanent pacemaker implantation (PPMI) is a known complication of the procedure that continues to occur more frequently in TAVR than in surgical aortic valve replacement. The reported incidence of PPMI after TAVR reached 27% in some studies,^1^ and this has been associated with increased length of stay (LOS), rehospitalizations, higher costs, and worse long-term outcomes. Efforts to decrease PPMI after TAVR have recently focused on achieving a more aortic implantation of the device through modified angiographic implantation views (i.e., cusp overlap technique) and conservative management of left-bundle branch block after the procedure.^2^^,^^3^ It is unclear whether such efforts have resulted in lower in-hospital PPMI rates or a shift toward outpatient PPMI after the index hospitalization. We used the Nationwide Readmissions Database (NRD), Healthcare Cost and Utilization Project (HCUP) to analyze post-TAVR PPMI rates from 2016 to 2018.

We identified our cohort, procedures, and outcomes using the International Classification of Diseases, 10th Revision, Clinical Modification (ICD-10-CM) and procedure (ICD-10-PCS) codes. After identifying patients with TAVR, we excluded those with missing data on in-hospital mortality and those with a PPM or implantable cardioverter defibrillator (ICD) history. The NRD includes patients from approximately 58% of hospitals in the United States, and therefore, national estimates were calculated to reflect numbers of admissions in the United States as per the HCUP recommendations. The NRD is a publicly available database with deidentified hospitalization records; therefore, institutional review board approval was not required. The outcomes of our study were PPMI during the initial hospitalization and readmission for PPMI. Analyses of national estimates were conducted using the appropriate weighting, stratifying, and clustering samples following HCUP regulations. Categorical variables are displayed as numbers and percentages. Continuous variables are summarized as medians and interquartile range (25th and 75th percentiles) and were compared using the Wilcoxon rank-sum test. All *p*-values are 2-sided with a significance threshold <0.05. All trend analyses were performed using the Poisson regression method. We performed multivariate logistic regression analysis to identify predictors of 30-day readmission for PPMI using the “Enter method” and variables based on background knowledge. Statistical analysis was performed using STATA software for Windows (version 17.0. College Station, TX: StataCorp LLC).

We identified 80,184 total hospitalizations for TAVR during the study period. After excluding records with missing data on in-hospital mortality and PPM or ICD history, our final cohort included 70,245 patients (national estimate of 126,794 admissions). Of those, 7848 underwent PPMI (national estimate of 14,038 records, 11.1%). There was a significant trend toward lower PPMI over the study period (12.3% in the first quarter of 2016 to 10.6% in the last quarter of 2018) (*p* trend <0.001) (Figure 1). Patients who had PPMI had longer LOS (4 [3-8] vs. 2 [2-4] days, *p* < 0.001) and higher cost of index hospitalization ($60,725 [$47,574-$76,683] vs. $45,109 [$35,560-$58,011], *p* < 0.001) than those who did not.

**Figure 1:**
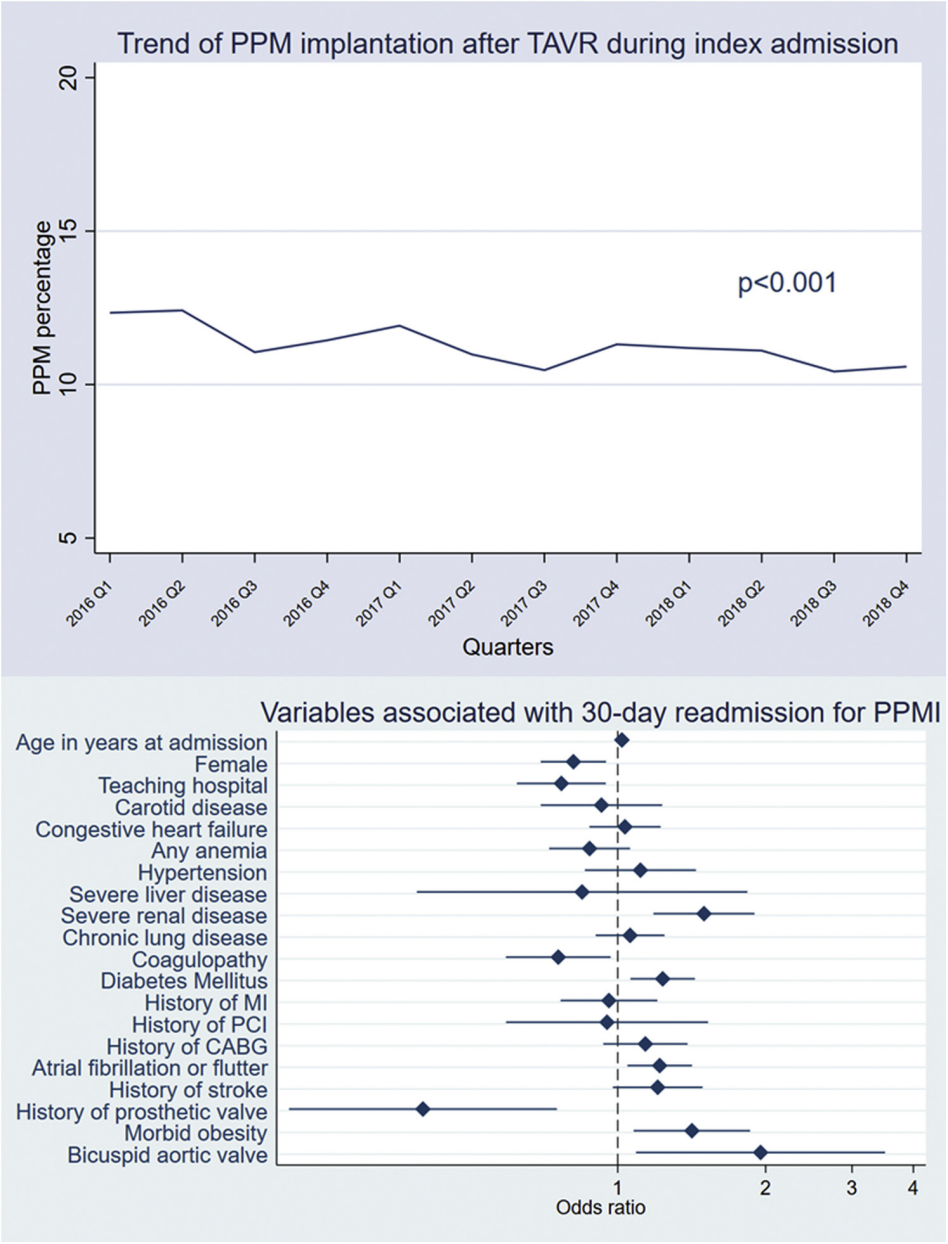
**Trends of in-hospital permanent pacemaker implantation rate after transcatheter aortic valve replacement and predictors of 30-day readmission for permanent pacemaker implantation after discharge**.

The 30-day readmission rate was calculated for patients discharged alive after TAVR and with no PPM or ICD history who did not receive PPM during the index hospitalization. A total of 6953 patients were readmitted (national estimate of 12,488 records, 12.4%). Eight hundred sixty-two patients were readmitted for PPMI (national estimate of 1602 records, 1.5%). There was no significant change in the trend of patients readmitted for PPMI from 2016 to 2018. The median time to readmission for PPM implantation was 4 [2-7] days. Bicuspid aortic valve, history of atrial fibrillation or flutter, morbid obesity, and severe renal disease were independently associated with 30-day readmission for PPMI, while female sex and having the procedure at a teaching hospital were protective against readmission for PPMI (Figure 1).

The main findings of our study of trends in pacemaker utilization after TAVR are (1) overall PPM implantation during the index hospitalization is decreasing, (2) patients who had PPM implantation had a higher LOS and cost of index hospitalization, (3) the 30-day readmission for PPM implantation remained constant at 1.5%, and (4) bicuspid aortic valve, history of atrial fibrillation or flutter, morbid obesity, and severe renal disease were important predictors of 30-day readmission for PPMI. Our study results are consistent with the recently published TVT registry analysis.^4^ From 2016 to 2018, the rates of in-hospital (11%, 9.8%, and 9.4%) and 30-day (13%, 11.8%, and 11.4%) PPMI were also decreasing. Importantly, the decrease in in-hospital PPMI was not offset by an increase in 30-day PPMI rates, which would signal a shift in timing rather than true reduction in PPM utilization.

A major limitation of administrative databases in the United States, including the NRD and the TVT registry, is the limited ability to capture mechanistic links that can account for these trends. Continuous device enhancements coupled with technical refinements and standardization of postprocedure pathways may account for these differences, but this requires further validation. Efforts should continue to focus on optimal patient selection for, and timing of, PPMI. Previous studies have shown that only 10% of patients with new post-TAVR left bundle branch block who received a PPM were PPM-dependent at 1 ​year.^5^ Although we identified important clinical predictors of 30-day readmission for PPMI, our analysis lacked imaging and electrocardiogram predictors and should be interpreted with caution.

## Ethics statement

The research reported has adhered to the relevant ethical guidelines. The NRD is a publicly available database with deidentified hospitalization records; therefore, institutional review board approval was not required.

## Funding

The authors have no funding to report.

## Disclosure statement

Santiago Garcia is a consultant for BSCI, Medtronic, Edwards Lifesciences, and Abbott Vascular. Dr Garcia has received institutional grant support from 10.13039/100006520Edwards Lifesciences, 10.13039/100004374Medtronic, BSCI, and 10.13039/100011949Abbott Vascular.

The other authors have nothing to disclose.
